# Characteristics of Kaposi sarcoma in HIV-infected patients

**DOI:** 10.1186/1471-2334-14-S4-P5

**Published:** 2014-05-29

**Authors:** Raluca Jipa, Oana Streinu-Cercel, Șerban Benea, Iulia Niculescu, Roxana Petre, Elisabeta Otilia Benea, Ruxandra Moroti, Cristina Popescu, Victoria Aramă, Adriana Hristea

**Affiliations:** 1 National Institute for Infectious Diseases “Prof. Dr. Matei Balş”, Bucharest, Romania; 2 Carol Davila University of Medicine and Pharmacy, Bucharest, Romania

## 

Objective: to describe clinical and laboratory characteristics; to assess predictors for death in HIV-infected patients with Kaposi sarcoma (KS).

We performed a retrospective study of HIV-infected patients diagnosed with KS in one infectious diseases hospital in Romania, between January 2008-November 2013. KS diagnosis was established on physical examination, skin biopsy, and for visceral involvement upper gastrointestinal endoscopy, bronchoscopy and computed tomography. KS was staged according to the AIDS Clinical Trials Group (ACTG) [1] and the Mitsuyasu classification system [2].

We identified 27 HIV-infected patients with KS. The median age was 42 years (IQR 34-52) and 18 (67%) were male. The median CD4 count at HIV diagnosis was 195 cells/cmm (IQR 55-313), while at KS diagnosis the median CD4 count was 101 cells/cmm (IQR 41-270). Eighteen (67%) patients had a CD4 count <200 cells/cmm. The median HIV viral load at the time of KS diagnosis was 120,000 copies/mL (IQR 316-328,522). HIV infection was diagnosed before KS in 19 patients (70%), with a median time between HIV and KS diagnosis of 7 months (IQR 0-58). The most frequent KS localization was the lower limb in 16 (59%) patients and 7 (26%) patients had disseminated KS. Oral, gastrointestinal and pulmonary involvements were seen in 10 (37%), 4 (15%) and 3 (11%) patients respectively. Concomitant opportunistic infections were diagnosed in 20 (74%), while other malignancies in 3 (12%) patients. According to the ACTG classification 16 (59%) patients had poor risk KS. Fifteen (56%), 6 (22%), 1 (4%) and 5 (18%) were in stage 1, 2, 3 and 4, respectively according to the Mitsuyasu classification. Six (22%) patients received specific KS treatment: three local radiotherapy and three systemic therapy (two with interferon; one with liposomal doxorubicin). The overall mortality was 41% with a median duration between KS diagnosis and death of 6 months (IQR 2-15). Gastrointestinal involvement (p=0.019), poor-risk KS in ACTG classification (p<0.001) and stage IV Mitsuyasu (p=0.006) were associated with death in univariate analysis.

The mortality rate in this study was high, due to poor immunological status, extended KS and high incidence of opportunistic infections, but also due to the lack of specific systemic treatment.
